# Korean Red Ginseng Extract Increases Apoptosis by Activation of the Noxa Pathway in Colorectal Cancer

**DOI:** 10.3390/nu11092026

**Published:** 2019-08-29

**Authors:** Yoon A. Jeong, Bo Ram Kim, Dae Young Kim, Soyeon Jeong, Yoo Jin Na, Jung Lim Kim, Hye Kyeong Yun, Bu Gyeom Kim, Seong Hye Park, Min Jee Jo, Sun Il Lee, Byung-Cheol Han, Dae-Hee Lee, Sang Cheul Oh

**Affiliations:** 1 Graduate School of Medicine, Korea University College of Medicine, Seoul 02841, Korea; 2 Department of Oncology, Korea University Guro Hospital, Korea University College of Medicine, Seoul 08308, Korea; 3 Department of Surgery, Korea University Guro Hospital, Korea University College of Medicine, Seoul 08308, Korea; 4 Korea Ginseng Research Institute, Korea Ginseng Corporation, Daejeon 34337, Korea

**Keywords:** endoplasmic reticulum stress, korean red ginseng, Noxa, reactive oxygen species

## Abstract

Background: Although the anticancer activity of Korean Red Ginseng (KRG) has been known in various cancers, the mechanism of KRG-induced apoptosis is unknown in colorectal cancer (CRC). In our study, we examined whether KRG induces apoptosis in CRC cells. Methods: In the cell viability assay, the concentration of the appropriate KRG extracts was fixed at 2.5 mg/mL in numerous CRC cells. This fixed concentration was in other experiments, and it was confirmed that the KRG extracts induce apoptosis in CRC cells. Results: We found that KRG induced Noxa activation and apoptosis and increased endoplasmic reticulum stress via reactive oxygen species production. This indicated that KRG efficiently enhanced cell death in CRC cells. Conclusion: Our results show that KRG can be used as a possible anticancer drug for patients with CRC

## 1. Introduction

Colorectal cancer (CRC) is the most commonly occurring cancer worldwide [1]. Treatment options for CRC usually include surgery, radiation therapy, and chemotherapy. Although many therapies have been developed, the 5 year survival rate of patients with CRC is still low [2]. Therefore, alternative treatments are required to achieve improved therapeutic efficacy.

**Figure 1:**
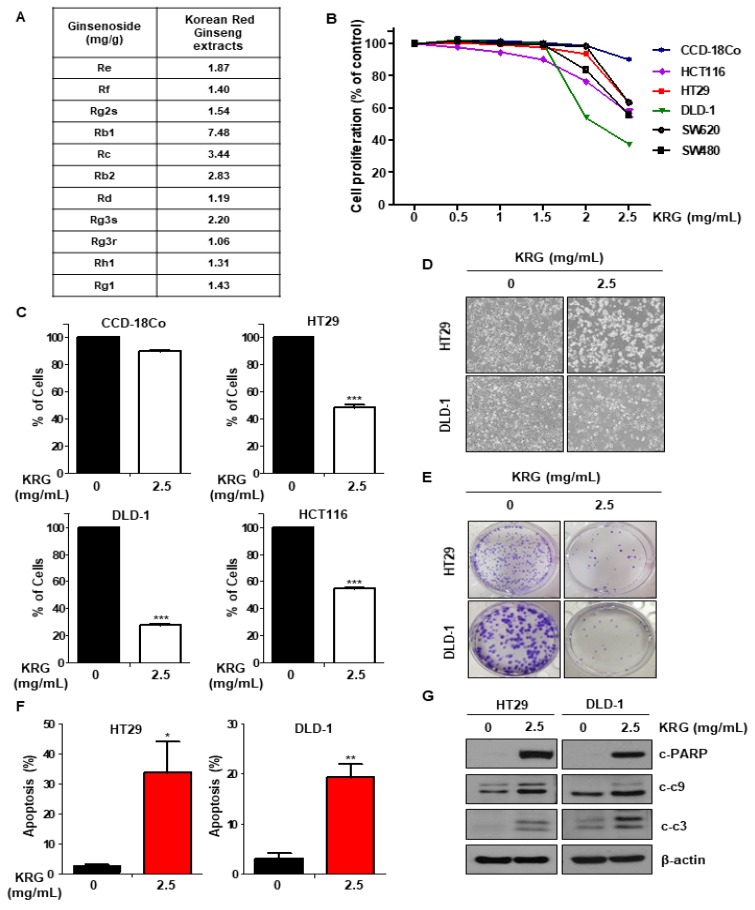
KRG extract reduces viability and induces apoptosis of human CRC cells. (**A**) Ginsenoside content (mg/g) of KRG extract. (**B**) The cell proliferation of normal colon cell line (CCD-18Co) and various CRC was detected by the MTT assay after treatment with 0–2.5 mg/mL KRG extract. (**C**) Cell proliferation of CRC and CCD-18Co cell lines was assessed by trypan blue staining after KRG extract treatment. Cells were incubated in the absence or presence of 2.5 mg/mL KRG extract for 48 h. (**D**) HT29 and DLD-1 cells were treated with 2.5 mg/mL KRG extract for 48 h, and the morphology of the cells was evaluated by light microscopy. Scale bar: 100 μm. (**E**) HT29 and DLD-1 cells were treated with 2.5 mg/mL KRG extract. After 2 weeks, the cells were stained with crystal violet and were photographed using a digital camera. (**F**) Levels of cleaved caspase-3, cleaved caspase-9, and cleaved PARP were detected by western blotting. (**G**) HT29 and DLD-1 cells were treated with 2.5 mg/mL KRG extract for 48h, stained with annexin V/PI, and assessed using FACS analysis.The data are shown as the mean of many repeated independent experiments. *** *p* < 0.001, ** *p* < 0.01, * *p* < 0.05. KRG: Korean Red Ginseng. CRC: colorectal cancer. FACS: fluorescence-activated cell sorting.

Herbal medicines have long been used for the prevention and treatment of various diseases, in the form of traditional treatments [3]. Herbs are widely used for therapeutic treatments, but among them, ginseng is the most widely used herb worldwide. Ginseng is usually divided into three types: fresh ginseng (less than 4 years), white ginseng (4–6 years, dried after peeling), and red ginseng (6 years, dried after steaming) [4]. Korean Red Ginseng (KRG) is a traditional herbal medicine that has been used to treat various diseases such as cancer, Alzheimer’s disease, anti-inflammatory diseases, and diabetes [5,6,7]. In previous reports, KRG has been shown to exert anticancer activity in various cancers [8,9]. KRG extracts contain the following major ginsenosides: Rb1, Rc, Rb2, Rg3s, RE, Rg2s, Rg1, Rf, Rh1, Rd, and Rg3r (Figure 1A). However, the mechanism of KRG-induced apoptosis is unknown in CRC cells.

Noxa is a pro-apoptotic protein, which encodes a Bcl-2 homology 3 (BH3)—only member of the Bcl-2 family. Noxa is translocated to the mitochondria, releases cytochrome c, and then induces apoptosis. Additionally, Noxa is reported to play a critical function in cell death through Bax-mediated mitochondrial dysfunction [10,11].

Endoplasmic reticulum (ER) stress is associated with cellular processes such as protein folding, synthesis, modification, and stress sensing [12,13]. It is related to the pathogenesis of various diseases, including Parkinson disease, neuronal damage, and Alzheimer’s disease [14]. Additionally, several signaling pathways are reported to be directly associated with ER stress-induced apoptosis [15]. According to previous reports, activation of Noxa is known to be regulated by ER stress. [16]. 

In this study, we investigated whether KRG induces cell apoptosis, and found that KRG treatment efficiently enhanced apoptosis in CRC cells. Here, we showed for the first time that KRG increases apoptosis via Noxa activation and the intrinsic apoptosis pathway. Furthermore, we found that KRG induces ER stress via the production of reactive oxygen species (ROS). Overall, our study suggests that KRG may serve as an attractive therapeutic drug for patients with CRC.

## 2. Materials and Methods

### 2.1. Cell Culture

Human CRC HT29, DLD-1, HCT116, SW480, and SW620, and human normal colon CCD-18Co cells were purchased from American Type Culture Collection (Manassas, VA, USA). Human CRC cells were cultured in RPMI 1640 medium from GenDEPOT (Katy, TX, USA) or McCoy’s 5A medium. CCD-18Co cells were cultured in Eagle’s minimal essential medium (American Type Culture Collection). All media contained 1% antibiotic–antimycotic (100 X; GenDEPOT) and 10% fetal bovine serum (Sigma-Aldrich, St. Louis, MO, USA). All cell lines were maintained in a 5% CO_2_ incubator at 37 °C.

### 2.2. Reagents and Antibodies

Korean Red Ginseng powder was kindly provided by the Korea Ginseng Corporation (Daejeon, Korea). The KRG powders were dissolved in distilled water. *N*-acetyl-l-cysteine (NAC) and Anti-β-actin antibody were purchased from Sigma-Aldrich (St. Louis, MO, USA). Anti-caspase-3, anti-caspase-9, anti-cleaved PARP, anti-BIM, anti-Noxa, anti-survivin, anti-IRE1α, anti-phospho IRE1α, anti-GRP94, anti-eIF2α, anti-phospho eIF2α, anti-ATF6, anti-PERK, anti-phospho PERK, anti-Bip anti-XBP1s, anti-ATF4, anti-SOD2, anti-SOD1, anti-catalase, anti-NOX4, and anti-NOX2 antibodies were purchased from Cell Signaling Technology (Danvers, MA, USA). Anti-Bak, anti-BAX, anti-Bcl-2, anti-SOD3, and anti-CHOP antibodies were purchased from Santa Cruz Biotechnology (Santa Cruz, CA, USA). The anti-mouse and Rabbit IgG HRP, the secondary antibodies, were purchased from Cell Signaling Technology (Danvers, MA, USA).

### 2.3. Cell Viability Assay

The proliferation of cells was examined using the 3-(4,5-dimethylthiazol-2-yl)-2,5-diphenyltetrazolium bromide (MTT) (Sigma-Aldrich) assay. The cells were seeded and incubated with MTT for 4 h. After 4hr incubation, the supernatant was removed, and the cells were solubilized in 150 μL dimethyl sulfoxide. Cell proliferation was detected by assessing the absorbance at 595 nm using a microplate reader. Additionally, cell counts were obtained by trypan blue (Amresco, Inc., Solon, OH, USA) staining. The cell pellets were resuspended in phosphate-buffered saline and stained using trypan blue. The number of live cells was counted with a hemocytometer.

### 2.4. Colony Formation Assay

The colony formation assay was used to assess cell proliferation. The cells were seeded in 60 Φ (60 × 15 mm) dishes. After incubation for 24 h, cell were subjected to KRG extract treatment for another 48 h. The cells were detached by trypsin (Trypsin-EDTA, GenDEPOT), and then seeded in 6 well plates and grown at 37 °C, in 5% CO_2_ incubator for colony formation. After 2 weeks, the cells were stained with crystal violet.

### 2.5. Western Blotting

Cells were harvested, and western blotting was carried out, as previously described [17]. The signals were detected with an X-ray film using electrochemiluminescence solution (DOGEN, Seoul, Korea).

### 2.6. Cell Apoptosis Assay by Flow Cytometry

The apoptosis of cell was assessed using the annexin V-propidium iodide (PI) apoptosis detection kit (BioBud, Cat. LS-02-100). Cells were incubated for 30 min at 4 °C in the dark, and then detected by flow cytometry.

### 2.7. Small Interfering RNA (siRNA)

Noxa siRNA, CHOP siRNA, and IRE1α siRNA were purchased from Santa Cruz Biotechnology. Cells were transfected using Lipofectamine RNAiMax reagent (Invitrogen), according to the manufacturer’s instructions.

### 2.8. Immunofluorescence Staining

Cells were seeded on glass coverslips and treated with KRG extract. After 48h, cells were fixed with 3.7% formaldehyde and permeabilized with 0.5% Triton X-100 for 15 min at room temperature. The cells were blocked for 1 h with 3% bovine serum albumin at 4 °C, and then incubated with primary antibodies overnight at 4 °C. Cells were then incubated with secondary antibodies in the dark. 4ʹ,6-diamidino-2-phenylindole (DAPI, Invitrogen, CA, USA), was used to stain the nuclei of cells. Stained cells were mounted (Vector Laboratories, Burlingame, CA, USA) and visualized using immunofluorescence microscopy.

### 2.9. ROS Measurement 

ROS levels were determined using carboxy-DCF-DA (2ʹ,7ʹ–dichlorofluorescin diacetate) (Thermo Fisher Scientific, Waltham, MA, USA). Cells were incubated for 30 min with 20 µM carboxy-DCF-DA, and then assessed by fluorescence-activated cell sorting (FACS) or a confocal microscope (Carl Zeiss AG, Oberkochen, Germany).

### 2.10. Quantitative Real-Time PCR

RNA was extracted using TRIzol reagent (TRI reagent, Molecular Research Center, OH, USA). Taqman probes (Thermo Fisher Scientific) were used for qRT-PCR to detect the mRNA expression of SOD3 (Hs00162090_m1) and GAPDH (Hs99999905_m1). GAPDH was used as the control.

### 2.11. Statistical Analysis

GraphPad InStat 6 software was used for all statistical analyses (GraphPad Software, Inc., La Jolla, CA, USA). Statistical data were analyzed using one-way analysis of variance, followed by Tukey’s post hoc tests. To determine the differences between the two groups, an unpaired *t*-test was performed, and a *p* value <0.05 was considered statistically significant.

## 3. Results

### 3.1. KRG Extract Inhibits the Viability and Increases the Apoptosis of CRC Cells 

We performed the MTT assay to determine cell proliferation following KRG extract treatment in CRC cell lines. KRG extract decreased the proliferation of CRC cells in a dose-dependent manner, but not the normal colon cell CCD-18Co (Figure 1B). Additionally, treatment with KRG extract enhanced apoptosis in CRC cell lines (HT29, HCT116, and DLD-1), as shown by trypan blue staining, but did not affect normal colon cell (CCD-18Co) (Figure 1C). We observed the morphology of HT29 and DLD-1 cells treated with KRG extract by light microscopy. The cell morphology treated with KRG extract was altered as compared with control cells (Figure 1D). The colony formation assay was performed to observe the long-term effect of KRG extract on cell survival. Cells treated with KRG extract showed inhibited colony formation and growth as compared with the control (Figure 1E). Additionally, FACS analysis following annexin V/PI staining confirmed that KRG extract is involved in apoptosis. As shown in Figure 1F, KRG extract induced apoptosis (Figure 1F), and the activity of cleaved caspase-3, caspase-9, and PARP was also increased (Figure 1G). These results indicated that treatment with KRG extract induces apoptosis in CRC cells.

### 3.2. KRG Extract Increases Apoptosis via Noxa Activation

**Figure 2:**
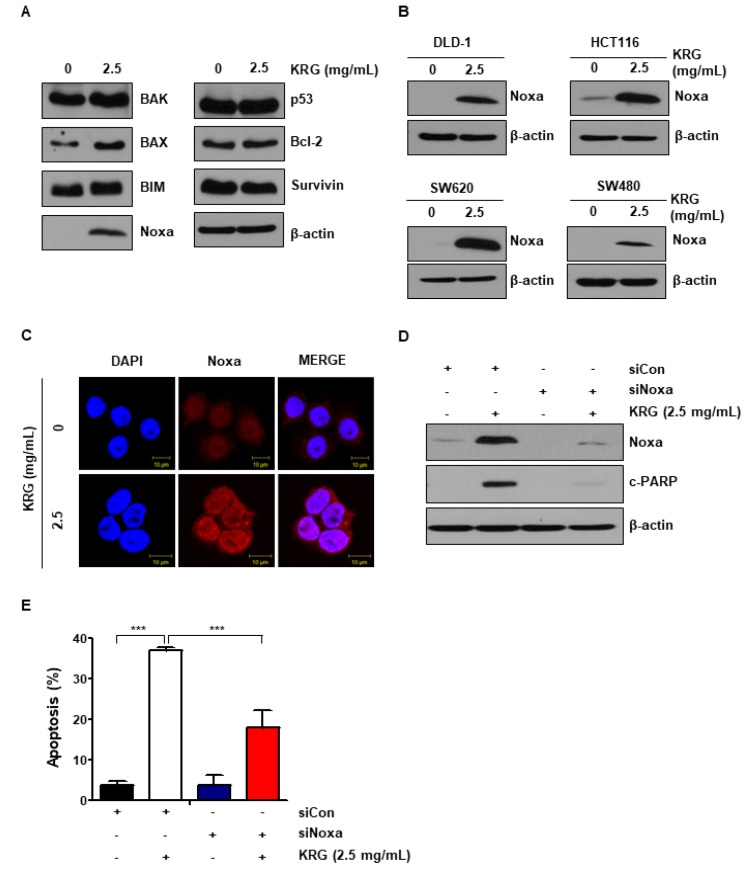
KRG extract enhanced apoptosis by regulating the expression of Noxa. (**A**) HT29 cells were treated with 2.5 mg/mL KRG extract for 48 h. The expression of pro-apoptotic proteins, such as BAX, Bak, Noxa, and BIM and anti-apoptotic proteins, such as Bcl-2 and survivin were detected by western blotting. (**B**) DLD-1, HCT116, SW620, and SW480 cells were treated with KRG extract (2.5 mg/mL) for 48 h. The expression of Noxa was confirmed by western blotting. (**C**) Noxa expression was observed by immunofluorescence and confocal microscopy. Scale bar: 10 μm. (**D**) HT29 cells were transfected with control or Noxa siRNA. And then cells were treated with 2.5 mg/mL KRG extract for 48 h. The expression of Noxa and cleaved PARP was analyzed by western blotting. (**E**) HT29 cells were transfected with control siRNA or Noxa siRNA. The cells were then treated with 2.5 mg/mL KRG extract for 48 h and stained with annexin V/PI. The rate of cell apoptosis was assessed by flow cytometry. The data are shown as the mean of many repeated independent experiments. *** *p* < 0.001.

We examined the level of anti-apoptotic and pro-apoptotic proteins to study the mechanisms of KRG extract. As shown in Figure 2A, we found that the expression of Noxa was significantly increased by treatment of KRG extract (Figure 2A). The increase of Noxa expression induced by KRG extract was also confirmed in HCT116, SW620, DLD-1, and SW480 cells (Figure 2B). Using immunofluorescence microscopy, we found that cells treated with KRG extract showed significantly higher expression of Noxa than control cells (Figure 2C). In order to determine whether the cell death induced by KRG extract was dependent on Noxa expression, cells were transfected with Noxa-specific siRNA. Knockdown of Noxa significantly reduced cleaved PARP level (Figure 2D) and apoptosis (Figure 2E). In other words, these data show that KRG extract increases apoptosis through activation of Noxa.

### 3.3. Cell Apoptosis Induced by KRG Extract Mediates the Activation of the ER Stress–Noxa Pathway

**Figure 3:**
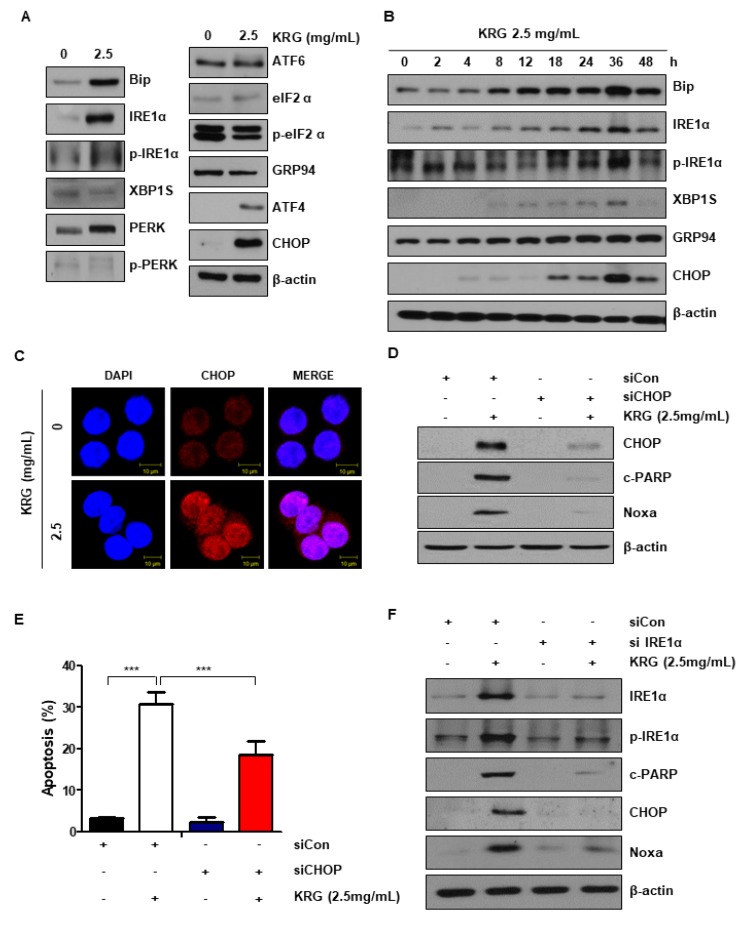
KRG extract induced the activation of ER stress. (**A**) HT29 cells were treated with KRG extract (2.5 mg/mL) for 24 h. The expression levels of Bip, IRE1α, p-IRE1α, XBP1s, PERK, p-PERK, ATF6, eIF2α, p-eIF2α, GRP94, ATF4, and CHOP (ER stress-related proteins) were observed by western blotting. (**B**) The level of the ER stress markers was measured by western blotting for the indicated times. (**C**) CHOP expression was assessed by immunofluorescence and confocal microscopy. Scale bar: 10 μm. (**D**) HT29 cells were transfected with control siRNA or CHOP siRNA. The cells were then treated with 2.5 mg/mL KRG extract for 48 h. The expression of CHOP, Noxa, and cleaved PARP, was analyzed by western blotting. (**E**) HT29 cells were transfected with control siRNA or CHOP siRNA. The cells were then treated with 2.5 mg/mL KRG extract for 48 h and stained with annexin V/PI. The rate of cell apoptosis was confirmed by flow cytometry. (**F**) HT29 cells were transfected with control siRNA or IRE1α siRNA. And then the cells were then treated with 2.5 mg/mL KRG extract for 48 h. The expression of IRE1α, p-IRE1α, CHOP, Noxa, and cleaved PARP was analyzed by western blotting. The data are shown as the mean of many repeated independent experiments. *** *p* < 0.001.

It has been indicated that ER stress is associated with cell apoptosis regulation [16,18]. We measured the level of ER stress-related proteins to determine whether KRG extract is involved in ER stress. The treatment of KRG extract dramatically increased the expression of p-IRE1α and CHOP (Figure 3A,B). The increase of CHOP expression was also confirmed by immunofluorescence (Figure 3C). To investigate the association between ER stress and cell apoptosis increased by KRG extract, cells were transfected with CHOP siRNA. As a result, CHOP knockdown reduced cleaved PARP, CHOP, and Noxa levels, indicating that Noxa expression can be blocked by CHOP knockdown (Figure 3D). CHOP knockdown also significantly reduced KRG extract-induced apoptosis, as shown by FACS analysis (Figure 3E). Additionally, knockdown of IRE1α reduced cleaved PARP, IRE1α, p-IRE1α, CHOP, and Noxa levels increased by KRG extract treatment (Figure 3F). These results confirm that KRG extract induces apoptosis via the IRE1α-CHOP-Noxa pathway.

### 3.4. KRG Extract Induces ROS Generation

**Figure 4:**
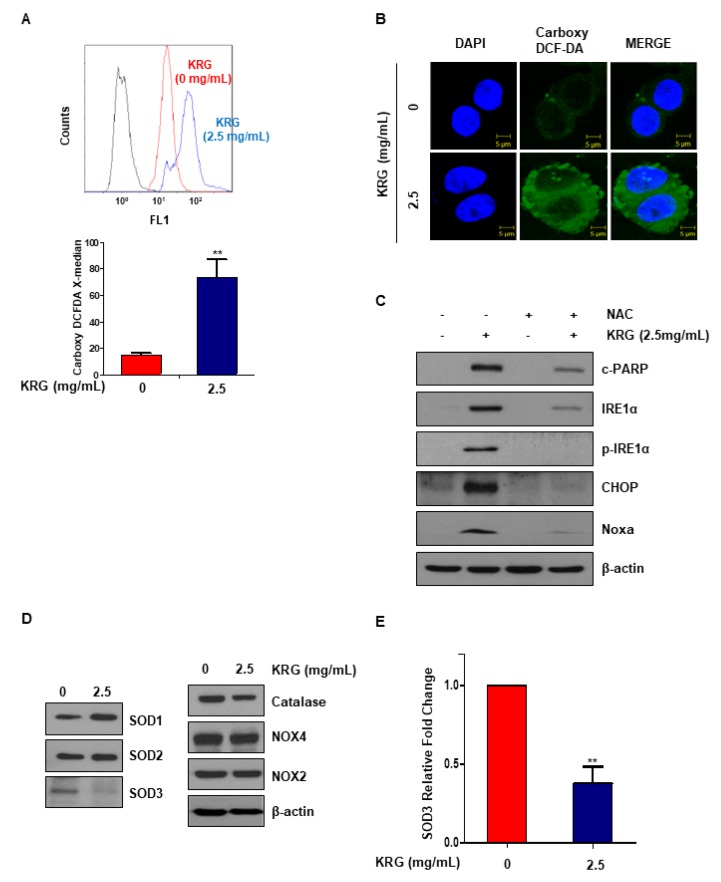
KRG extract induced ROS production. (**A**) HT29 cells were treated with KRG extract (2.5 mg/mL) for 48 h. At 30 min prior to harvest, the cells were treated with carboxy-DCF-DA (10 µM). The levels of ROS were determined by carboxy-DCF-DA staining and confirmed by flow cytometry. (**B**) ROS generation was assessed by immunofluorescence and confocal microscopy. Scale bar: 10 μm. (**C**) HT29 cells were pretreated with 10 mM NAC for 2 h, and then treated with 2.5 mg/mL KRG extract for 48 h. The levels of Noxa, CHOP, IRE1α, p-IRE1α, and cleaved PARP were detected by western blotting. (**D**) HT29 cells were treated with 2.5 mg/mL KRG extract for 48 h. The expression of SOD1, SOD2, SOD3, catalase, NOX2, and NOX4 were measured by western blotting. **(E)** The mRNA level of SOD3 was determined by qRT-PCR. The data are shown as the mean of many repeated independent experiments. ** *p* < 0.01. SOD: superoxide dismutase.

Because ROS and ER stress are related, it was thought that cell apoptosis induced by KRG extract might induce ER stress via ROS generation. We measured ROS production by carboxy-DCF-DA. KRG extract treatment significantly increased ROS production (Figure 4A). Immunofluorescence microscopy was also performed to examine the expression of carboxy-DCF-DA. These results confirmed that treatment with KRG extract induced ROS generation (Figure 4B). *N*-acetyl-l-cysteine, antioxidant, was administered to further investigate whether ROS affects KRG extract-mediated cell apoptosis. As a result, NAC inhibited the apoptosis induced by KRG extract (Figure 4C). We examined the expression of antioxidant proteins (SOD1, SOD2, SOD3, and catalase) and superoxide radical-forming enzymes (NOX2 and NOX4), which are ROS-related factors, in order to examine whether KRG extract regulates ROS production. SOD3 protein expression was decreased, and the level of *SOD3* mRNA was also confirmed (Figure 4D,E). Our results suggest that KRG extract induces apoptosis through increasing ROS–ER stress via inhibition of *SOD3* mRNA.

## 4. Discussion

In previous studies, KRG has been known to have anticancer effects in CRC cells [19,20]. However, its mechanism has not yet been well elucidated. In our study, we found that treatment of KRG extract induced cell death in CRC cells, but not in normal epithelial primary cells (Figure 1B,C). Because KRG is a natural product, it is safe and less toxic. Therefore, we suggest KRG as an alternative anticancer drug. 

Apoptosis is the process of programmed cell death that can be regulated by anti-apoptotic proteins and pro-apoptotic proteins [21]. We examined the change of pro-apoptotic and anti-apoptotic proteins expression in CRC cells treated with KRG extract. For the first time, we found that KRG extract exhibited pro-apoptotic effects via activation of Noxa in CRC cell lines (DLD-1, HCT116, SW620, and SW480) (Figure 2B). Noxa is regulated by the tumor suppressor gene p53 and is known to be associated with P53-mediated apoptosis [22]. As shown in Fig. 2A, the level of p53 protein was not changed after treatment of KRG extract. These results indicated that KRG extract increased p53-independent Noxa activation. 

Since Noxa activation was p53-independent, we evaluated ER stress, which is known to affect Noxa expression [16]. ER stress is a cellular process that affects protein folding, synthesis, and trafficking [23,24]. ER stress is known to cause the activation of Noxa via the PERK/ATF4/CHOP, IRE/JNK, or IRE1α/CHOP pathway [25,26]. We found that the ER stress increased by KRG extract increases Noxa expression through the IRE1α/CHOP pathway (Figure 3A,B). Treatment with KRG extract increased the phosphorylation of JNK, but the increase in apoptosis and CHOP and Noxa expression induced by KRG extract was not changed by treatment with JNK inhibitor (data not shown). These results confirmed that KRG extract induces apoptosis via the IRE1α-CHOP-Noxa pathway. ROS plays a key function in regulating physiological functions, such as proliferation, differentiation, cell cycle, cell death, and migration [27]. Especially, ROS is associated with tumor initiation and cancer progression. Since cancer cells have higher ROS levels than normal cells, they are susceptible to acute oxidative stress. In contrast, excessive ROS can cause necrosis or apoptosis of cancer cells, which is important in cancer therapy [28,29]. In previous studies, induction of ROS has been reported to cause apoptosis by activating ER stress [30]. Consistent with these reports, our results showed that KRG extract induces ROS generation (Figure 4A,B). Because NAC, a scavenger of ROS, is an aminothiol and synthetic precursor of cysteine and Glutathione, it is considered to be an antioxidant [31]. NAC plays a role in protecting against carcinogenesis and DNA damage through its antioxidant activity in cancer. Additionally, we confirmed that NAC attenuates the KRG extract-mediated increase of ER stress and apoptosis (Figure 4C). ROS generation is reported to be controlled by SOD (superoxide dismutase) enzymes [32]. SOD enzymes are distinguished based on metal ion cofactors and localization. Eukaryotes express cytoplasmic Cu/ZnSOD (SOD1), mitochondrial MnSOD (SOD2), and extracellular Cu/ZnSOD (SOD3) [33]. We investigated the expression of SOD enzymes and NOXs to identify how KRG extract regulates ROS production. Treatment of KRG extract dramatically decreased the level of SOD3 protein and mRNA (Figure 4D,E). These results indicated that KRG extract induces ROS generation via inhibition of SOD3 transcription. 

## 5. Conclusions

**Figure 5:**
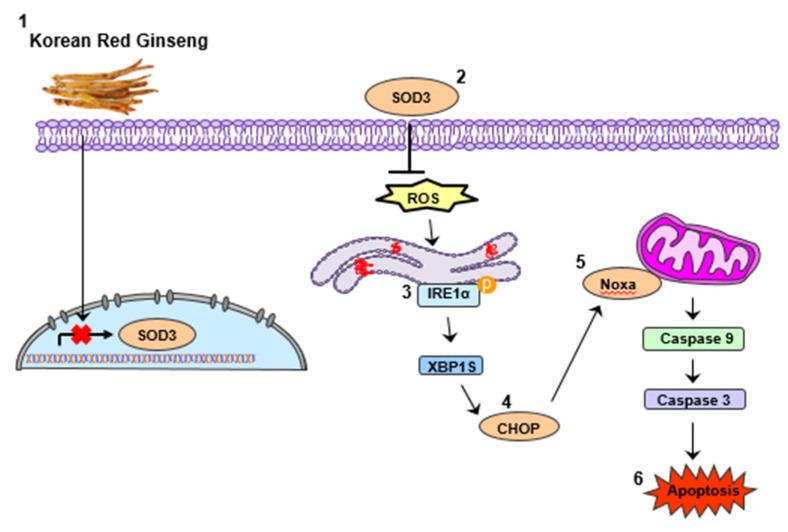
Schematic diagram for apoptosis induced by KRG extract.

In summary, we found that treatment with KRG extract increases apoptosis via Noxa activation in CRC cells, and Noxa activation is enhanced by induction of ROS–ER stress (Figure 5). Taken together, our study suggests that KRG extract could serve as a therapeutic drug for patients with CRC.
